# Damage Evolution of RC Beams Under Simultaneous Reinforcement Corrosion and Sustained Load

**DOI:** 10.3390/ma12040627

**Published:** 2019-02-20

**Authors:** Jiansheng Shen, Xi Gao, Bo Li, Kun Du, Ruoyu Jin, Wei Chen, Yidong Xu

**Affiliations:** 1 Ningbo Institute of Technology, Zhejiang University, Ningbo 315100, China; sjs@nit.zju.edu.cn (J.S.); kstg2868134@163.com (K.D.); chenw@nit.zju.edu.cn (W.C.); 2 School of Mathematical Sciences, University of Nottingham Ningbo China, Ningbo 315100, China; xi.gao@nottingham.edu.cn; 3 Department of Civil Engineering, University of Nottingham Ningbo China, Ningbo 315100, China; 4 School of Civil Engineering, Chongqing Jiaotong University, Chongqing 400074, China; 5 School of Environment and Technology, University of Brighton, Brighton BN2 4GJ, UK; R.Jin@brighton.ac.uk

**Keywords:** damage evolution, reinforced concrete beam, reinforcement corrosion, sustained load, finite element model

## Abstract

To accurately obtain the performance of concrete structures in coastal regions, it is necessary to correctly understand the damage evolution law of reinforced concrete (RC) members under real working conditions. In this paper, four RC beams, subjected to different levels of corrosion and sustained load, are first tested. Reinforcement corrosion coupled with sustained load increases the number and width of cracks at the soffit of beams but decreases their loading capacities. Crack width of the corroded beam under 50% of designed load is two times of that under 30% of designed load. Residual loading capacities of the corroded beams subjected to 30% and 50% of designed load are 87.5% and 81.8% of the control beam. A finite element model is developed for the corroded RC beams. Due to less confinement, concrete below and at the sides of reinforcements is subjected to a higher stress, compared to concrete above the reinforcements. Corrosion expansion of reinforcements is successfully modelled by a temperature-filed method, as it properly simulates the damage evolution of the corroded RC beams. As a result, concrete cracking, caused by the reinforcement corrosion, is well captured. Coupling reinforcement corrosion with sustained load significantly increases the damage level in RC beams, particularly for those subjected to a high sustained load. The whole damage evolution process of concrete cracking due to corrosion expansion under the coupling effect of sustained loading and environment can be simulated, thus providing a reference for the durability evaluation, life prediction, and numerical simulation of concrete structure.

## 1. Introduction

The durability of reinforced concrete (RC) is one of the most concerning issues for infrastructure around the world. Tang et al. [1] reviewed recent research activities on the durability of concrete and recommended developing a new approach for accurately estimating the durability service life of RC structures. Reinforcement corrosion is regarded as a crucial factor in causing the deterioration of RC structures [2]. In order to accurately obtain the service characteristics of concrete structures in coastal regions, many studies have been devoted to investigations on the performance of RC structural members, subjected to reinforcement corrosion. With the assumption of uniform reinforcement corrosion, Zhang et al. [3] presented the test results of two corroded RC beams subjected to 14 years and 23 years chloride exposure, and proposed a relationship between area loss of reinforcements and crack width of beams. Khan et al. [4] investigated the cracking development of corroded RC beams after exposing to 26 years corrosion in the laboratory and estimated the evolution of reinforcement corrosion inside the RC beams. Results found that steel cross-sectional loss in the stirrups has no relationship with the crack width of longitudinal cracks. Their test results were also compared with predictions of the Rodriguez model [5], the Vidal model [6], and the Zhang model [3]. Gu et al. [7] conducted 19 reinforced concrete slabs to study the non-uniform corrosion characteristics and mechanical properties of corroded reinforcement under chloride attack. A new non-uniform corrosion factor, R, was used to analyze the reliability of RC beams. Murthy et al. [8] studied the performance of RC beams that were retrofitted with a thin ultra-high strength concrete (UHSC) strip. The results showed that the damaged RC beams can be successfully rehabilitated by using a thin precast UHSC strip.

The numerical simulation of reinforcement corrosion is an efficient tool to estimate the performance of the corroded RC beams. Xu [9] discussed the influence of pitting geometry on the tensile behavior of reinforcement by using finite element analysis (FEA). Based on a multi-phase and multi-species modelling method, Liu et al. [10] and Mao et al. [11] investigated different ionic transport features in concrete composite. Jiang et al. [12] presented a mesoscopic numerical model to investigate the mechanism of chloride diffusion under freeze–thaw cycles, which can reflect the coupled effect of the freeze–thaw process and the chloride diffusion process at time scale. Xi et al. [13] proposed a meso-scale fracture model for the reinforcements by incorporating the influence of aggregates, cement paste, and interfacial transmission zone. The proposed corrosion model was able to simulate the non-uniform corrosion of reinforcements in the middle and corner of beam section. Yang et al. [14] introduced a cohesive crack model to simulation crack initiation and propagation in the corroded RC structural members, which was successfully used to predict the crack width of the corroded RC structures. Abddelatif et al. [15] proposed a 3D chemo-hygro-thermo-mechanical model to simulate the corrosion of reinforcements in the lap spliced joints in RC structures. The proposed 3D model was further verified by the test results from the literature. Zhu and Zi [16] proposed a two-dimensional corrosion model for reinforcements, which couples the effects of chloride ions, carbonation, electrochemical reaction, and corrosion-induced damage. It was successfully used to predict the non-uniform distribution of corrosion production, expansion stress, and crack propagation in the RC beams. Richard et al. [17] and Al-Osta et al. [18] investigated the damage of corroded RC beams by using different corroded bars and concrete interface models. Shayanfar and Safiey [19] proposed an algorithmic for producing the tension-stiffening curve of RC elements, taking into account factors such as the rate of steel bar corrosion, bond-slip behavior, concrete cover, and amount of reinforcement. Since numerous structures have been constructed and retrofitted by various types of new materials and techniques, the dynamic performance and shear capacity of RC structural members has been an important issue [20,21]. Based on a cohesive finite element technique, Weinberg and Khosravani [22] conducted a numerical fracture simulation about the dynamic properties of Ultra-High-Performance Concrete (UHPC) made with low-silica content. Mohammad et al. [23] adopts the extended finite element method to simulate the crack initiation and propagation of UHPC material under dynamic Brazilian test. Li et al. [21] proposed a numerical model for investigating the shear behavior of bolted side-plated (BSP) beams. The OpenSees software was employed for the numerical simulation.

In the coastal regions, reinforcement corrosion, coupled with sustained load, has been recognized as the main issue affecting the durability of RC structures. Dong et al. [24] investigated the flexural behavior of RC beams under simultaneous sustained load and steel corrosion. Chloride ions were electro-migrated into the RC beams, followed with a DC current under wetting and drying cycles. Test results indicate that coupling sustained load with reinforcement corrosion leads to more severe and faster cracking damage in the RC beams. Li et al. [25] tested the RC beams under simultaneous loading and reinforcement corrosion and indicated that sustained load accelerates the crack propagation along the longitudinal direction of beam. A higher sustained loading level and longer corrosion exposure are prone to cause the brittle failure of RC beams. Similarly, Zhang et al. [26] tested RC beams under simultaneous sustained load and chloride ingress and illustrated that reinforcement corrosion has a marginal influence on the distribution of transverse cracks in RC beams. In addition, reinforcement corrosion in RC beams shall be properly considered, as it may exceed creep effect at a relatively low corrosion level. Zhang and Zhao [27] compared the structural performance of nature aggregate and recycled aggregate RC beams subjected to the coupled sustained load and reinforcement corrosion. It was found that the recycled aggregate RC beams had more severe damage than the nature aggregate RC beams. Hou et al. [28] investigated the corrosion behavior of reinforced concrete/ultra-high toughness cementitious composite (RC/UHTCC) beams under sustained loading and shrinkage cracking. The results indicated that the coupled effect of sustained loading can further aggravate the degradation in flexural capacity of corroded RC/UHTCC beams.

Many existing studies have been conducted to experimentally investigate the structural performance of RC beams subjected to simultaneous reinforcement corrosion and sustained load. However, there are limited studies focusing on the damage evaluation of the RC beams under combined reinforcement corrosion and sustained loads, particularly for numerical simulation of corroded RC beams. The authors of this study proposed a finite element model for the RC beams under coupled reinforcement corrosion and sustained load in which the elasto-plasticity model was adopted for concrete [29]. Thus, the damage evolution of corroded RC beams cannot be simulated. In this paper, the concrete damage plasticity (CDP) model was employed to simulate the damage evolution of RC beams under coupled reinforcement corrosion and sustained loads. Reinforcement corrosion is simulated by the thermal expansion of reinforcement under a designed temperature field. Meanwhile, an external load is applied to the RC beams. Focus is given to the damage of concrete around the corroded reinforcement in the RC beams under different levels of simulated corrosion expansion and sustained load. In order to verify the results of numerical simulation, the accelerated corrosion test, under coupled action of chloride attack and static loading, was conducted. The cracking behavior and static performance of RC beams with different corrosion degree were evaluated.

## 2. Experimental Program 

### 2.1. Specimens

**Figure 1:**
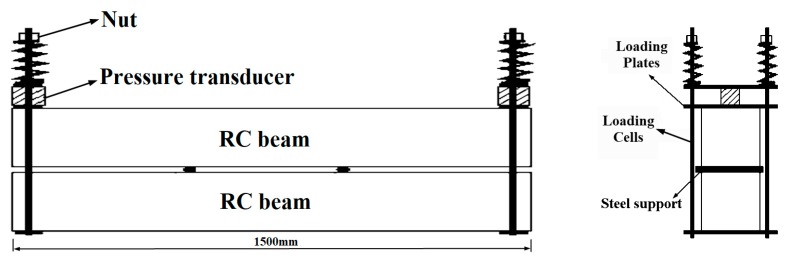
Schematic illustrations of the loading frame.

**Figure 2:**
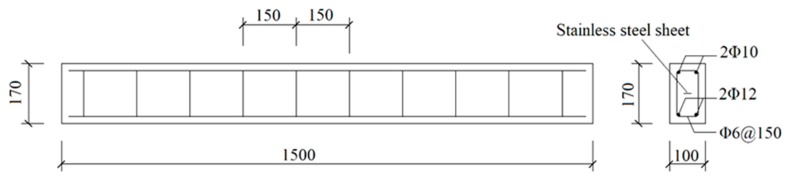
Details of the reinforced concrete beam.

Four RC beams were cast by the C40 concrete. The span of each RC beam was 1500 mm. In order to reflect the coupled effect of chloride ingress and static loading on RC beams, the specimens were loaded during the accelerated corrosion test by using a self-equilibrium loading frame, as shown in Figure 1. The sustained loads were applied by spring and nut, in which case, the dimension of the RC beams cannot be too large. In order to facilitate loading and ensure a certain thickness of concrete cover, the RC beam has a cross-section of 100 mm × 170 mm with the concrete cover of 20 mm. Two T12 rebars (HRB335) and two R10 (HPB235) rebars were used as bottom and top longitudinal reinforcements, respectively. HPB235 round steel rebars with a diameter of 6 mm were adopted as links with a spacing of 150 mm. It is worth noting that a stainless-steel belt, with the dimension of 10 mm × 1460 mm × 0.2 mm, was embedded in the center of the beam, which served as the cathode during the accelerated corrosion. The dimension and details of RC beam are shown in Figure 2. In the concrete mix formulation, ordinary Portland cement 42.5, river sands, and 20 mm crushed coarse aggregates are adopted. The measured 28-day cubic compressive strength of concrete is 42.5 MPa. 

### 2.2. Accelerated Corrosion Test

Accelerated corrosion of RC beams were conducted through chloride penetration and wet–dry cycles. The beams were first cured in a fog room with a temperature of 20 ± 2 °C and relative humidity of 95% for 28 days, which is a standard curing scheme in accordance with Chinese standard GB/T 500081-2002 [30]. Afterwards, the beams were subjected to an accelerated corrosion process of reinforcements and different levels of sustained load. According to the corrosion condition and sustained loads, four RC beams, including control beam without corrosion (i.e., specimen B), three corroded beams subjected to 0 (i.e., specimen BC), 30% (i.e., specimen BCL-30), and 50% (i.e., specimen BCL-50) of designed loading capacity, were constructed and tested as shown in Table 1.

**Figure 3:**
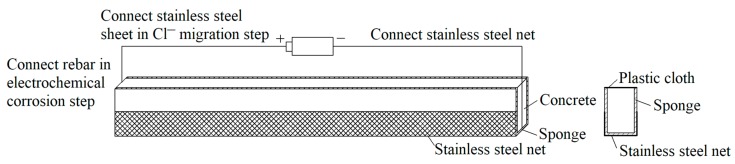
Schematic illustration of accelerated corrosion test of RC beam under chloride ingress.

In RC structural members, reinforcement corrosion usually occurs in localized non-uniform form under the chloride attack. A new accelerated corrosion test method, consisting of the electro-migration step and the wet–dry cycle step, has been adopted in this study [31,32]. In the first step, a sponge soaked with 5% NaCl solution covers on RC beams, followed with wrapping by a stainless-steel mesh and a plastic paper. The saturated sponge is kept for 30 h to keep the concrete moist. Subsequently, the stainless-steel belt embedded inside the RC beam and stainless-steel mesh outside the RC beam are connected to the anode and cathode of a 30V direct current power source, respectively. The chloride ions are migrated into concrete cover under the action of the electric field. In the second step, a wet–dry cycle is applied to the RC beams immediately after the electro-migration process. Each wet–dry cycle includes a 3-day drying process, followed by a 4-day wetting process. A constant current density of 200 μA/cm^2^ is applied to accelerate corrosion process during the wetting process. Figure 3 shows a schematic of the accelerated corrosion test of an RC beam under chloride ingress. 

**Figure 4:**
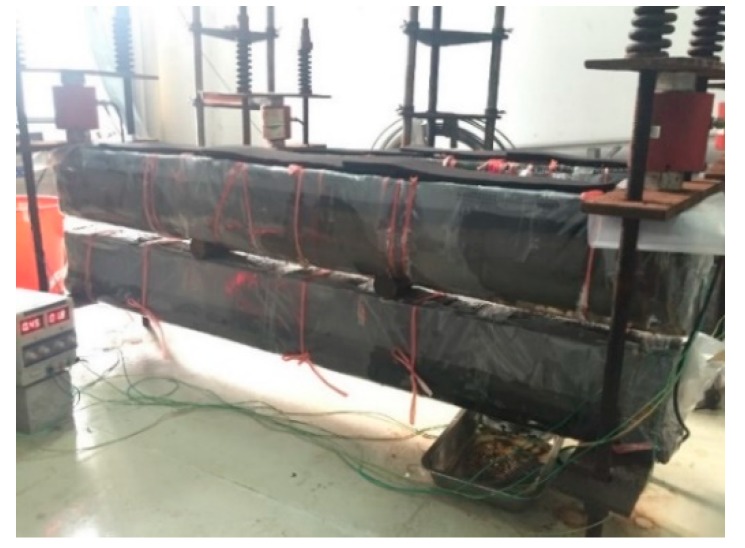
RC beam under accelerated corrosion and sustained load.

For the corroded RC beams subjected a sustained load, a constant load was applied by using the self-equilibrium loading frame consisting of loading cells, loading nuts, springs, and loading plates, as shown in Figure 4. Owing to the relaxation of loading springs, the applied loads will be reducing in a couple of days. In order to keep the applied loads constant, the loading springs were adjusted during the acceleration corrosion process. 

### 2.3. Loading Scheme

**Figure 5:**
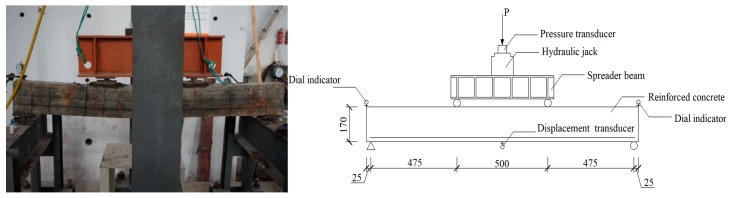
Test setup for the corroded beam (unit: mm).

A four-point bending test of the corroded RC beams was conducted in accordance with Chinese standards GB/T 50152-2012 [33]. Figure 5 shows the test setup for the beams. The beam is simply supported and is loaded through a loading spreader beam. Displacement transducers are installed at both ends and mid-span of the beam. A loading jack with 300 kN loading capacity is used to apply the bending moment. The step loading method was adopted in the experiment to stabilize the relationship between load and deformation. After each stage of loading, the load was held for 10 min, followed with the measurement of the displacements at the mid span and each support of the RC beam. Thus, the mid span deflection of RC beam was calculated as follows.

*f_m_* = *f*_3_ − ( *f*_1_ + *f*_2_)/2 (1)

where, *f_m_* is the mid span deflection of the RC beam; *f*_1_ and *f*_2_ are the displacements at each support of the RC beam, respectively; *f*_3_ is the displacements at the mid span of the RC beam.

## 3. Experimental Results and Discussion

### 3.1. Cracking Behavior of RC Corroded Beams

**Figure 6:**
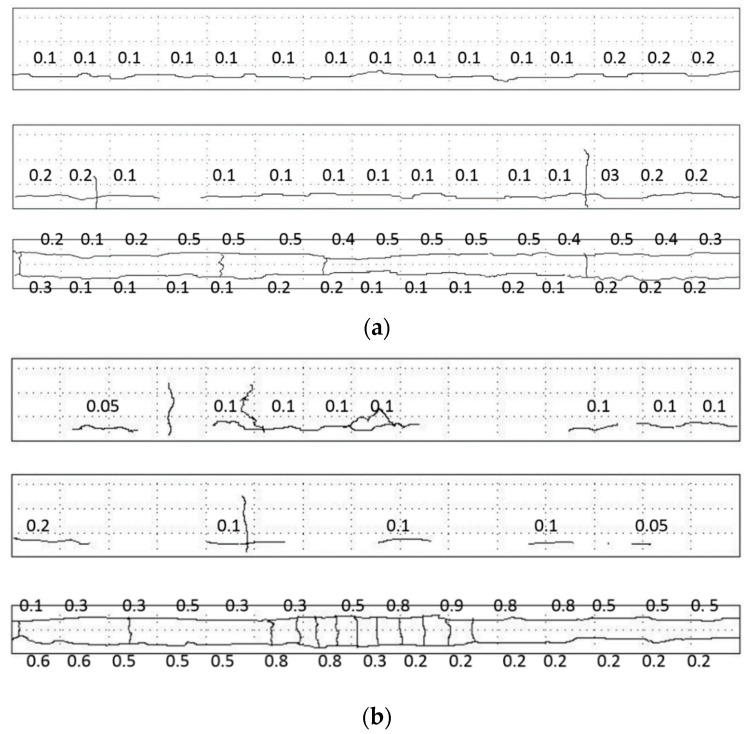

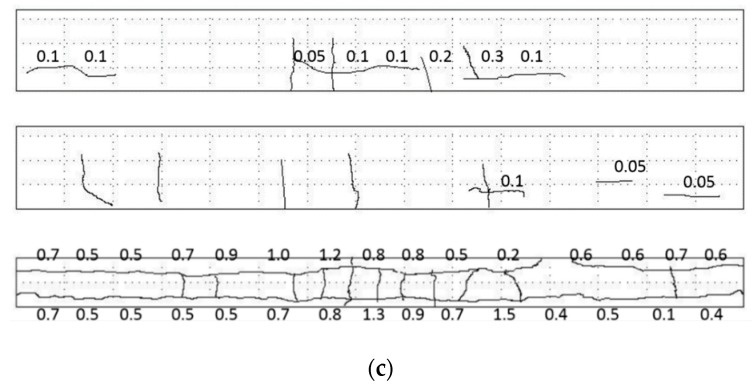
Cracking behavior of the corroded beams under (**a**) 0, (**b**) 30%, and (**c**) 50% of designed load (unit: mm).

Cracking behavior of RC beams, after being exposed to various levels of reinforcement corrosion, is shown in Figure 6. For the corroded beam subjected to chloride ingression only, cracks mainly distribute along the longitudinal direction on the sides and soffit of beam. The continuous cracks form on both sides of the beam along the longitudinal reinforcements. There are several transverse cracks only on the sides and bottom of the beam. Applying a sustained load, coupled with reinforcement corrosion, evidently alters the cracking behavior of RC beams. As the sustained load increases, longitudinal cracks on both sides of beams are reduced in terms of number and width, but with an increase of transverse cracks. Similarly, the number and width of cracks on the bottom of beams increase with the sustained load. This is mainly attributed to the higher tensile stress at the bottom of the corroded beam, which is caused by the corrosion expansion of reinforcements. Corrosion products around the reinforcements would press the surrounding concrete, inducing additional stresses in the concrete. However, the tensile stress of concrete at the sides of the beam is decreased due to the bending effect. Therefore, there are more cracks on the bottom of beam and less cracks on the sides of the beam. 

The width of cracks on the bottom of the beam increases with the sustained load. For the beam without sustained load, the cracks have the almost same width along the beam. As the sustained load increases, cracks are longer in the pure bending zone than those at both ends of the beam. Cracks at the bottom of the beam are much wider than those at the sides of the beam. For instance, the maximum width of crack at the bottom of beam is 1.5 mm, which is 67% wider than that for the beam under 30% of designed load and is 200% of the corroded beam without sustained load. 

### 3.2. Loading Capacities of the Corroded RC Beams

**Figure 7:**
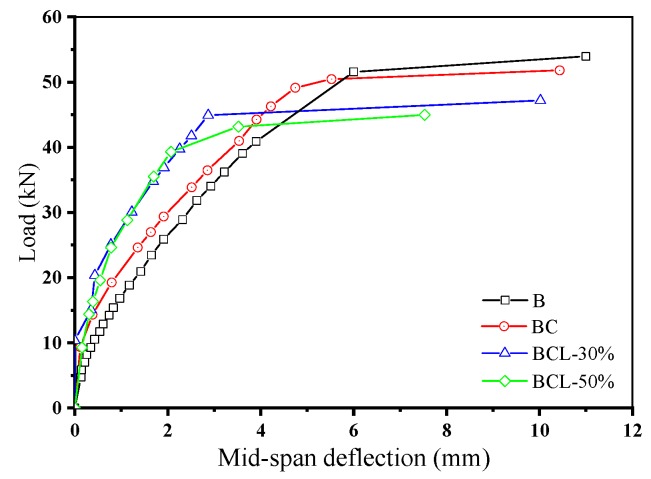
Load-mid span deflection relationships of the corroded beams.

Figure 7 shows the residual load–deflection relationships of the corroded beams under four-point bending. Test results of flexural test of four corroded beams are summarized in Table 2. As seen in Figure 7, the initial stiffness of corroded beams is higher than that of the beams without corrosion (i.e., specimen B). It indicates that the corrosion of reinforcements would enhance the initial flexural stiffness of RC beams. Moreover, applying sustained load would further increase the initial flexural stiffness of corroded beams. The initial stiffness of specimens BCL-30 and BCL-50 are obviously higher than that of the beams subjected to reinforcement corrosion only (i.e., specimen BC). Jin and Wang [32] conducted the experimental study on mechanics behaviors of corroded reinforced concrete beams. The result also shows that the initial stiffness of corroded beams is higher than that of the non-corroded beam. The enhancement of flexural stiffness of corroded beams is mainly attributed to the fact that the tensile stress is carried by the reinforcement at the cracks, which cannot be effectively transmitted to the concrete. 

Loading capacities of corroded beams decrease significantly as compared to the control beam without corrosion. Coupling sustained load with reinforcement corrosion further decreases the loading capacity of the corroded beams. As seen in Table 2, the loading capacities of corroded beams under 0%, 30%, and 50% of designed load is 96.1%, 87.5%, and 81.8% of that of beams without reinforcement corrosion. As the load is mainly taken by the longitudinal reinforcement, corrosion of reinforcement would obviously decrease the loading capacities of corroded beams due to the reduction in cross-section of reinforcements. Besides, considerable research work has been carried out on the bond strength between concrete and corroded reinforcement. The results show that severe corrosion significantly reduced steel/concrete bond strength [34,35]. Therefore, the deterioration of bond between the corroded reinforcements and concrete would be another reason for the reduction of loading capacity of corroded beams.

## 4. Simulation Results and Discussion

### 4.1. Concrete Plastic Damage Model

**Figure 8:**
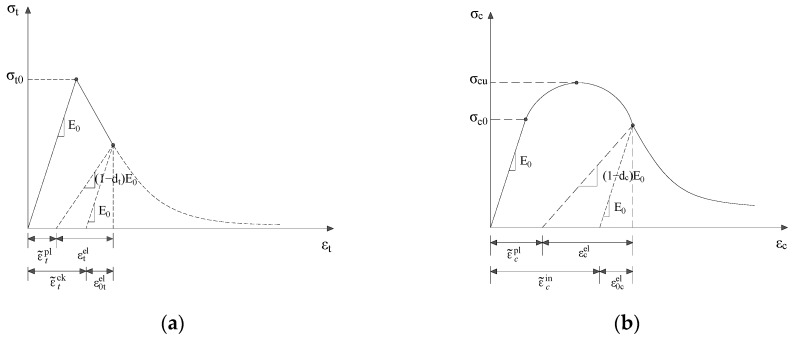
Constitutive model of concrete under (**a**) tension and (**b**) compression.

The concrete damage plasticity (CDP) model built in ABAQUS is adopted in this study, which employs concepts of isotropic damaged elasticity in combination with isotropic tensile and compressive plasticity to represent the inelastic behavior of concrete. The CDP model is capable of simulating loading cases of concrete subjected to monotonic, cyclic, and/or dynamic loading under low confining pressures [36]. In the cases of uniaxial tension and compression, assumptions are made that the two main failure mechanisms are tensile cracking and compressive crushing of concrete, as shown in Figure 8 [37]. Here, subscripts t and c represent tensile and compressive, respectively. Under uniaxial tension, the stress–strain relationship of the concrete material is purely elastic until the failure stress σ_t0_ is reached, where micro-cracks in the concrete material start emerging. Beyond the failure stress, the formation of micro-cracks becomes significant associated with a softening stress–strain response of the material. Under uniaxial compression, the response is linear until the value of initial yield stress σ_c0_ is reached. In the plastic regime the response is typically characterized by stress hardening followed by strain softening beyond the failure stress σ_cu_. Under both loading situations, the concrete material is modelled by the CDP model, which utilises two equivalent plastic strains, ${\tilde{\varepsilon }}_{c}^{pl}$ and ${\tilde{\varepsilon }}_{t}^{pl}$ to control the evolution of the yield and failure surface.

When the concrete is unloaded from any point on the strain softening branch of the stress–strain curves, the unloading response exhibits a weakened stiffness slope, indicating that the elastic stiffness of the material has been damaged. The damaged elastic stiffness is characterized by two damage indices, as shown in Equation (2).

(2) $$d_{t}=d_{t}({\tilde{\varepsilon }}_{t}^{pl},\theta ,f_{i});\;0\leq d_{t}\leq 1,\;d_{c}=d_{c}({\tilde{\varepsilon }}_{c}^{pl},\theta ,f_{i});\;0\leq d_{c}\leq 1$$

where *d_t_* and *d_c_* represent tensile and compressive damage index, respectively; θ stands for the temperature, and *f_i_* (*i* = 1, 2,…) represent other field variables involved.

Damaged stress–strain relations under compression and tension are characterized using damage indices, as in the following Equation (3).

(3) $$\sigma_{t}=(1-d_{t})E_{0}(\varepsilon_{t}-{\tilde{\varepsilon }}_{t}^{pl})\sigma_{c}=(1-d_{c})E_{0}(\varepsilon_{c}-{\tilde{\varepsilon }}_{c}^{pl})$$

where *E*_0_ is the initial elastic stiffness of the concrete.

Under uniaxial tension, the post-failure behavior for cracked concrete is modelled with tension stiffening, which is defined by means of a post-failure stress–strain relation. In RC member, the post-failure behavior is specified by defining the post-failure stress as a function of the cracking strain. Definition of the cracking strain is given in Equation (4).

(4) $${\tilde{\varepsilon }}_{t}^{ck}=\varepsilon_{t}-\varepsilon_{0t}^{el}$$

where $\varepsilon_{0t}^{el}=\sigma_{t}/E_{0}$ as shown in Figure 8. 

According to the tensile damage curve, $d_{t}-{\tilde{\varepsilon }}_{t}^{ck}$, the cracking strain can be converted to plastic strain as follows

(5) $${\tilde{\varepsilon }}_{t}^{pl}={\tilde{\varepsilon }}_{t}^{ck}-\frac{d_{t}}{\left(1-d_{t}\right)}\frac{\sigma_{t}}{E_{0}}$$

Under uniaxial compression, the stress–strain behavior of plain concrete outside the elastic regime needs to be defined. This is done by inputting the compressive stress as function of the compressive inelastic strain, ${\tilde{\varepsilon }}_{c}^{in}$. Strain-softening happens once the stress–strain curve becomes beyond the ultimate stress. The compressive inelastic strain is defined as the total strain minus the elastic strain corresponding to the undamaged material as follows

(6) $${\tilde{\varepsilon }}_{c}^{in}=\varepsilon_{c}-\varepsilon_{0c}^{el}$$

where, $\varepsilon_{0c}^{el}=\sigma_{c}/E_{0}$, as shown in Figure 8b.

Using the defined compressive damage curve, $d_{c}-{\tilde{\varepsilon }}_{c}^{in}$, the compressive inelastic strain can be converted to compressive plastic strain as follows.

(7) $${\tilde{\varepsilon }}_{c}^{pl}={\tilde{\varepsilon }}_{c}^{in}-\frac{d_{c}}{\left(1-d_{c}\right)}\frac{\sigma_{c}}{E_{0}}$$

### 4.2. Finite Element Model of RC Beams

**Figure 9:**
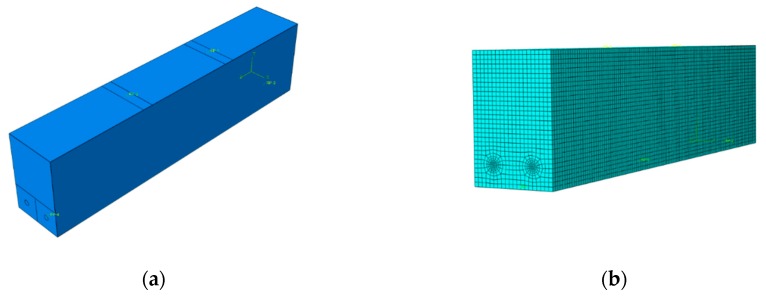
Finite element model of RC beam: (**a**) boundary conditions, and (**b**) meshed model.

The dimension of the FE model for RC beam is 100 mm × 150 mm × 600 mm with a cover thickness of 20 mm as shown in Figure 9a. Two T10 reinforcements are embedded in the beam as the bottom reinforcements. Damage indices were calculated following the damage evolution equations given in the Chinese Standard GB50010-2010 ‘Code for Design of Concrete Structures’ and converted to plastic damage as defined in ABAQUS using Equations (5) and (7). The material parameters used in the numerical simulation are given in Table 3 [38].

Static loads were applied to the top surface of the model over a certain width to avoid stress concentration. The two reference points on the top surface were coupled with the loading area so that loading could be applied through the reference points, then uniformly over the beam. Similarly, two reference points were placed on each side of the model to apply boundary conditions.

Considering the complexity of the stress in the area surrounding the steel, a finer mesh was employed. Therefore, the number of mesh seed for the steel and the reference line on the right is 5, while it is 5 for the rest part of the beam. The mesh was produced using the built-in sweep technique, in which mesh is first generated on the left-hand side of the model and extruded along the beam to mesh the whole beam, as shown in Figure 9b. The mesh is created using hexahedral elements C3D8R [29].

### 4.3. Application of Sustained Load and Reinforcement Corrosion

The load applied to the beam is four-point bending, so that the middle section of the beam is under pure bending. One end of the beam is fixed while the other end is simply supported. Boundary conditions are applied through the two reference points placed at the bottom of the two ends of the beam, while the loading is applied in the form of displacement through the two reference points in the middle.

There are various ways to simulate the corrosion expansion loading [39,40]. For two-dimensional models, the non-uniform corrosion of reinforcement is often simulated by applying non-uniform stress to the holes on the model representing reinforcement corrosion. However, for three-dimensional models, such application is not suitable as the reinforcements need to sustain loads. In order to simulate non-uniform corrosion expansion in three-dimensional model, the thermal expansion method is applied in this study. The linear expansion coefficient of the steel reinforcement is set to be 1.2 × 10^−5^/°C and the initial temperature is 0 °C. It is assumed that the corrosion is uniformly formed along the reinforcement, and the cohesive damage between the concrete and the reinforcement is neglected.

**Figure 10:**
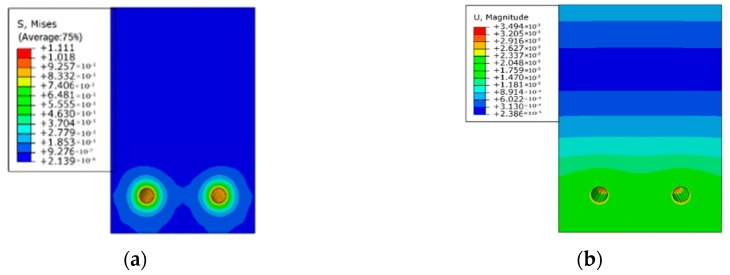
Thermal expansion of reinforcements-induced (**a**) stress, and (**b**) strain.

As seen in Figure 10, when the reinforcement is heated, the deformation tends to develop more significantly towards the bottom of the beam as a result of a concrete cover at the bottom. In reality, the thin concrete cover at the bottom of beam allows the air media to penetrate to the beam easier, which elevates the corrosion of reinforcement. Although the mechanism of the corrosion is different from the experimental study, they have similar consequences. Therefore, the thermal expansion method is validated to be used for simulating corrosion of reinforcements. Detailed simulation cases of sustained load and reinforcement corrosion for RC beams are tabulated in Table 4.

### 4.4. Damage Evolution of RC Beams under Reinforcement Corrosion and Sustained Load

**Figure 11:**
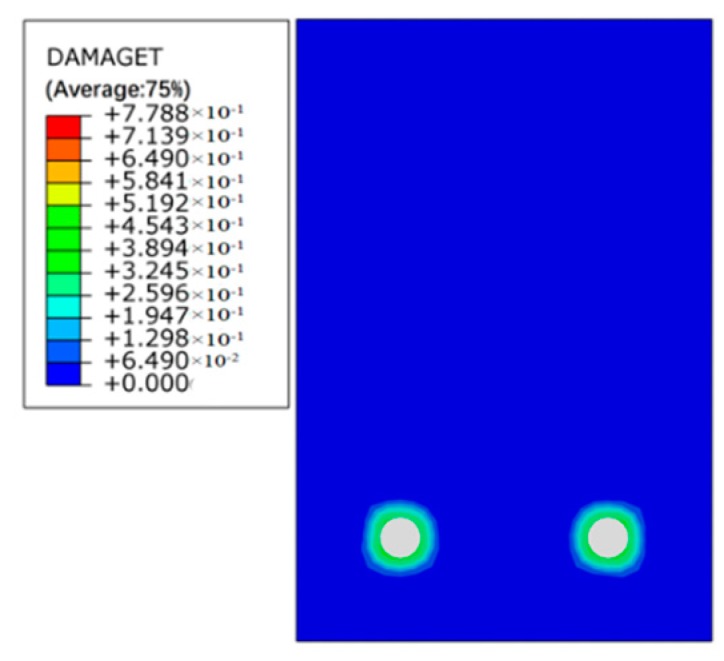
Damage index of mid-span beam section subject to reinforcement corrosion only (condition: L0-C20).

**Figure 12:**
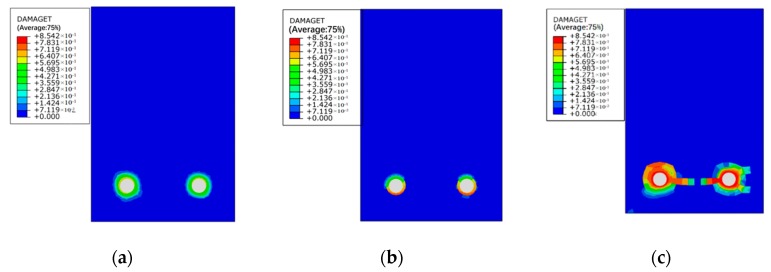
Damage indexes of mid-span beam sections under low level of reinforcement corrosion: (**a**) L1-C10, (**b**) L2-C10, and (**c**) L4-C10.

**Figure 13:**
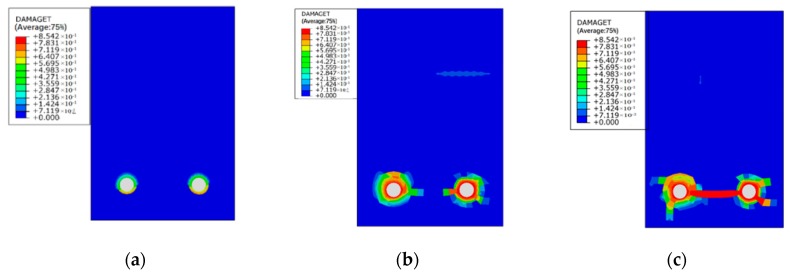
Damage indexes of mid-span beam sections under high level of reinforcement corrosion: (**a**) L1-C20, (**b**) L2-C20, and (**c**) L4-C20.

Figure 11, Figure 12 and Figure 13 show the damage evolutions on the cross-sections of the beams under different sustained load and reinforcement corrosion. For the beam subjected to reinforcement corrosion only, damage is primarily observed in the concrete–steel interface area while negligible damage occurs in the concrete, as can be seen in Figure 11. With increasing the sustained load, the damaged area in the cross-section grows constantly, and eventually emerges with the neighboring damaged area as shown in Figure 12 and Figure 13. The numerical results agree well with those in Xu et al. [41]. Besides, although under the same sustained load, there are obvious differences in the damage caused by different levels of corrosion expansion. A much more severe damage of concrete is caused when a larger corrosion expansion is applied, indicating that the coupling effect of the corrosion expansion and the sustained load intensifies the damage in the corroded beam.

**Figure 14:**
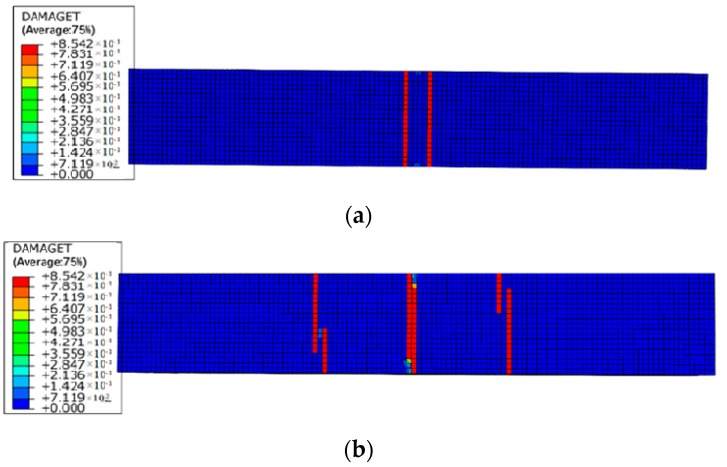

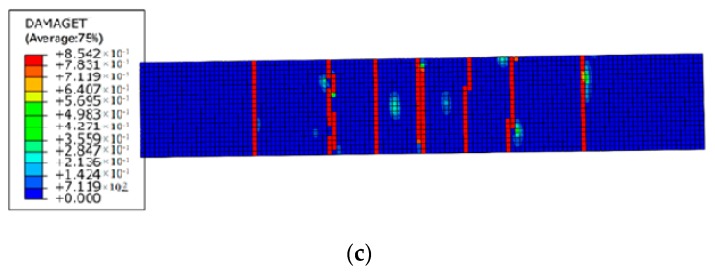
Damage indexes of RC beams under loading case of (**a**) L1-C10, (**b**) L2-C10, and (**c**) L4-C10.

**Figure 15:**
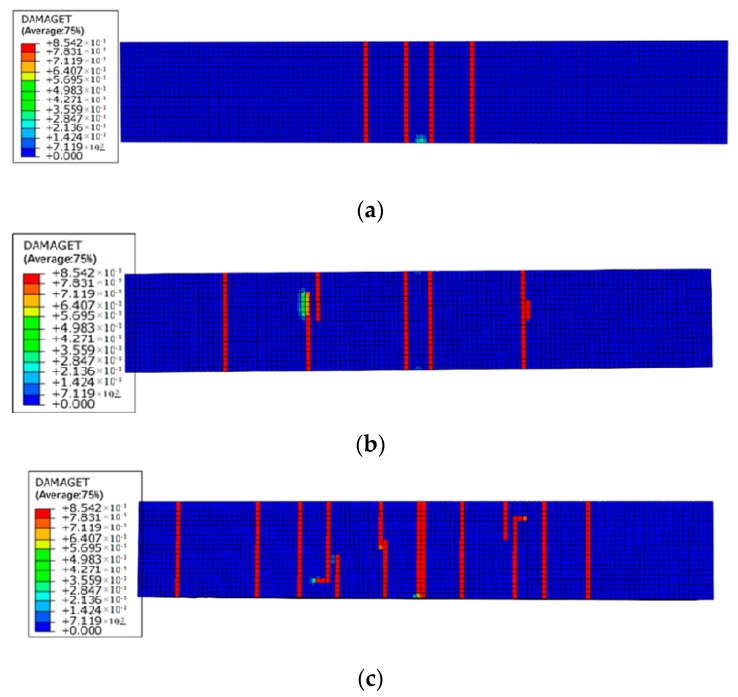
Damage indexes of RC beams under loading case of (**a**) L1-C20, (**b**) L2-C20, and (**c**) L4-C20.

Figure 14 and Figure 15 illustrate the damage evolution at the bottom of the beams under different levels of reinforcement corrosion and sustained load. As the sustained load and corrosion expansion increase, more cracks form in the bending area at the bottom of the beam. The bottom surface of the beam becomes more vulnerable to cracking formation under the coupling effect of the bending force and corrosion expansion force. As is shown in Figure 5 and Figure 15, the number of cracks at the bottom of RC beams increases generally with increased sustained load, which makes the corrosive medium easier to reach the surface of reinforcement. The corrosion damage of RC beams is prone to be more severe. The development of cracks in the axial direction is in harmony with the experimental results, which validates the numerical method in this paper.

**Figure 16:**
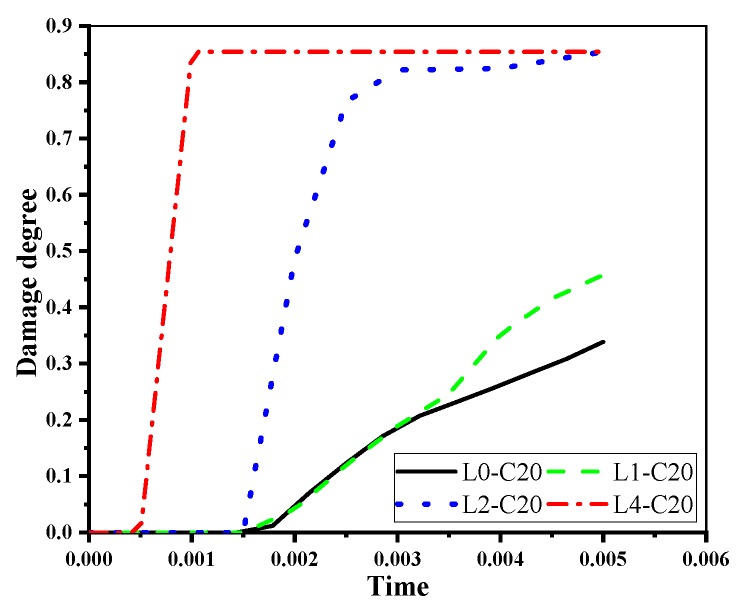
Damage evolution of the corroded beams under different levels of sustained loads.

Figure 16 shows the influence of the sustained load on the damage evolution of RC beams. When the sustained load is slight, the initial damage is chiefly controlled by the corrosion expansion. Therefore, the crack onset time of L0-C20, L1-C20, and L2-C20 are almost the same; when the sustained load becomes more significant, the initial damage is affected by both the sustained load and corrosion expansion. Therefore, the initial crack onset in L4-C20 is followed by quicker damage evolution and faster development and expansion of the cracks.

## 5. Conclusions

Damage evolution of RC beams under simultaneous reinforcement corrosion and sustained load is investigated in this paper. An experimental study of four RC beams, subjected to various levels of corrosion and loads, is first conducted. A finite element analysis is subsequently performed to simulate the corroded beams under sustained load and corrosion. Based on the experimental and simulation results, the following conclusions can be drawn.

1. Reinforcement corrosion, coupled with sustained load, changes the cracking behavior of RC beams. Increasing the sustained load reduces the number of cracks on the side of the beam but increases the number and width of cracks on the bottom of the beam. The crack width in the corroded beam under 50% of designed load is 67% higher than that under 30% of designed load.
2. Reinforcement corrosion, coupled with sustained load, decreases the residual loading capacities of RC beams, but increases their initial stiffness. Comparing to the control beam, the loading capacity of the corroded beams subjected to 0, 30%, and 50% of designed load is decreased by 3.9%, 12.5%, and 19.2%, respectively.
3. Stresses caused by the corrosion expansion of reinforcement are successfully simulated by the temperature filed method. With the influence of concrete cover, concrete stresses at both the sides and the bottom of the beam are higher than that above the reinforcement. This is consistent with the actual corrosion expansion of reinforcements in the RC beams.
4. Parametrical studies indicate that coupling reinforcement corrosion with sustained load significantly increases the damage level in the beams. Increasing sustained loading intensifies the concrete damage around the reinforcement, particularly for the concrete below the reinforcements. Further increases in the sustained load aggregates the concrete damage until concrete cracking.

## Figures and Tables

**Table 1:** Details of reinforced concrete (RC) beams under reinforcement corrosion and sustained load.

Specimen	Sustained Load	Accelerated Corrosion Process	Description
B	N/A	N/A	Control beam
BC	N/A	Step 1: electo-migration for 30 h Step 2: 6 wet–dry cycles under a constant current density of 200 μA/cm^2^, each cycle includes 3-day drying followed with 4-day wetting.	Non-sustained load
BCL-30	30% M_u_		Sustained load
BCL-50	50% M_u_		Sustained load
Note: M_u_ is the ultimate loading capacity of the RC beam.			

**Table 2:** Summary of load capacities of the corroded beams.

Specimen	B	BC	BCL-30	BCL-50
Load capacity (kN)	53.9	51.8	47.2	44.1
Reduction as compared to specimen B	N/A	3.90%	12.43%	18.18%
Reduction as compared to specimen BC	N/A	N/A	8.88%	14.86%

**Table 3:** Parameters for ABAQUS material definition of concrete and reinforcement.

Parameters for Concrete	Taken Value	Parameters for Reinforcement	Taken value
Modulus of elasticity	32.62 GPa	Modulus of elasticity	190 GPa
Poisson’s ratio	0.2	Poisson’s ratio	0.3

**Table 4:** Cases of sustained load and reinforcement corrosion for RC beams.

Specimen	Sustained Load (mm)	Corrosion Expansion (°C)
L0-C20	0	20
L1-C10	0.0625	10
L1-C20	0.0625	20
L2-C10	0.125	10
L2-C20	0.125	20
L4-C10	0.25	10
L4-C20	0.25	20
